# Immune profile of an atypical EAE model in marmoset monkeys immunized with recombinant human myelin oligodendrocyte glycoprotein in incomplete Freund’s adjuvant

**DOI:** 10.1186/s12974-015-0378-5

**Published:** 2015-09-17

**Authors:** S. Anwar Jagessar, Nicole Heijmans, Erwin L A Blezer, Jan Bauer, Robert Weissert, Bert A. ‘t Hart

**Affiliations:** Department of Immunobiology, Biomedical Primate Research Centre, P.O. Box 3306, 2280 GH Rijswijk, The Netherlands; ErasMS Centre, Erasmus Medical Center, Rotterdam, The Netherlands; Image Sciences Institute, University Medical Center Utrecht, Utrecht, The Netherlands; Center for Brain Research, Medical University of Vienna, Vienna, Austria; Department of Neurology, University of Regensburg, Regensburg, Germany; Department of Neuroscience, University of Groningen, Groningen, The Netherlands

**Keywords:** Common marmoset, Autoimmunity, Multiple sclerosis, Refinement, MOG

## Abstract

**Background:**

Experimental autoimmune encephalomyelitis (EAE) in the common marmoset monkey (*Callithrix jacchus*) is a relevant preclinical model for translational research into immunopathogenic mechanisms operating in multiple sclerosis (MS). Prior studies showed a core pathogenic role of T and B cells specific for myelin oligodendrocyte glycoprotein (MOG). However, in those studies, the quality of the response against MOG epitopes was strongly biased by bacterial antigens in the complete Freund’s adjuvant (CFA), in which the immunizing recombinant human (rh) MOG protein had been formulated. In response to the need of a more refined EAE model, we have tested whether disease could also be induced with rhMOG in incomplete Freund’s adjuvant (IFA).

**Method:**

Marmosets were immunized with rhMOG emulsified in IFA in the dorsal skin. Monkeys that did not develop neurological deficit were given booster immunizations at 28-day interval with the same antigen preparation. In a second experiment, three marmoset twin pairs were sensitized against MOG peptides in IFA to study a possibility for suppressive activity towards pathogenic T cells directed against the encephalitogenic epitope MOG40-48.

**Results:**

Despite the absence of strong danger signals in the rhMOG/IFA inoculum, all monkeys developed clinically evident EAE symptoms. Moreover, in all monkeys, demyelinated lesions were present in the white matter and in two cases also in the cortical grey matter. Immune profiling at height of the disease showed a dominant T cell response against the overlapping peptides 14–36 and 24–46, but reactivity against the pathogenically most relevant peptide 34–56 was conspicuously absent. In the second experiment, there was an indication for a possible suppressive mechanism.

**Conclusions:**

Immunization of marmoset monkeys with rhMOG in IFA elicits clinical EAE in all animals. Moreover, rhMOG contains pathogenic and regulatory epitopes, but the pathogenic hierarchy of rhMOG epitopes is strongly influenced by the adjuvant in which the protein is formulated.

**Electronic supplementary material:**

The online version of this article (doi:10.1186/s12974-015-0378-5) contains supplementary material, which is available to authorized users.

## Background

Experimental autoimmune encephalomyelitis (EAE) in the common marmoset (*Callithrix jacchus*), a small-bodied Neotropical primate, is a valid preclinical model of the human neuroinflammatory disease multiple sclerosis (MS). The genetic and immunological proximity of the marmoset to humans makes the model particularly useful for translational research into immunopathogenic mechanisms as a basis for new therapies (for review, [1, 2]).

To set up the EAE model, we essentially replicated well-established mouse models of EAE, the chronic relapsing model in Biozzi ABH mice for example [3]. Randomly selected marmosets were immunized with human myelin isolated from the brain of a MS patient, which was formulated with complete Freund’s adjuvant (CFA) [4]. We showed that, just like in the mouse model, myelin oligodendrocyte glycoprotein (MOG) is an essential myelin component for the induction of progressive disease [5, 6]. In subsequent studies, we determined the critical T cell epitopes in an EAE model induced with recombinant human MOG (rhMOG) formulated with CFA. We noticed that the initiation and progression of the disease are mediated by different pathogenic mechanisms (reviewed in [1]). Briefly, EAE initiation involves the activation of MHC class II/Caja-DRB*W1201-restricted T helper (Th) 1 cells specific for the epitope MOG24-36 [7]. Consistent with this classical mouse EAE-like paradigm, we observed a strong clinical effect of prophylactic treatment with ustekinumab, a humanized monoclonal antibody (mAb) against the joint p40 subunit of interleukins (IL)-12 and -23 [8]. We discovered that full clinical development of EAE involves transition to a previously unknown pathogenic mechanism, mediated by MHC class I/Caja-E-restricted effector memory cytotoxic T cells (CTL) specific for the epitope MOG40-48 [9]. Later, Zaguia et al. reported that similar pathogenic cells could be found in MS lesions [10].

The observation that full clinical EAE development could be induced by immunizing marmosets with a synthetic peptide representing residues 34 to 56 of rhMOG (peptide MOG34-56) formulated with incomplete Freund’s adjuvant (IFA), i.e. a formulation lacking the normally requisite danger signals, illustrates the remarkably high reactivity of the CTL [11]. Of note, immunization of immunologically naïve, SPF-bred, Biozzi ABH or C57BL/6 mice with the same formulation had no detectable effect [11]. We observed that the CTL in marmosets may be recruited from a repertoire of anti-viral T cells [12] and that B cells infected with the EBV-related marmoset γ-herpes virus CalHV3 are involved in the activation of the CTL [13, 14]. Finally, we observed that the CTL induce demyelination without the support of anti-MOG antibodies, probably by killing oligodendrocytes [15], as was also observed in MS lesions [10].

The marmoset EAE model induced with MOG34-56 in IFA represents a high level of refinement as the detrimental consequences of CFA are avoided. However, the model is insufficiently complete for serving as a relevant preclinical model of MS as the induction of demyelinating antibodies by autoreactive B cells is lacking. A characteristic of such pathogenic antibodies is that they bind conformational epitopes present in the rhMOG protein [16]. This led us to test whether EAE can be induced by immunization of marmosets with full-length rhMOG protein in IFA. We observed that this is indeed the case [17].

Here, we report the immunological characterization of the marmoset EAE model induced with rhMOG/IFA. Antibodies binding ELISA-plate bound rhMOG and MOG expressed in native human myelin are detectable in the plasma of rhMOG immunized monkeys. Remarkably, we observed only activation of T cells against the MOG24-36 epitope, while T cell reactivity against the pathogenically more important epitope MOG40-48 was absent. It is of note that the two epitopes are juxta-positioned in an evolutionary conserved region, residues 20–50, of the extracellular MOG domain. Physiological relevance of the strong evolutionary conservation of this region across mammalian species (ranging from the naked mole rat to the human) is likely, albeit poorly understood [18].

We asked whether the absent reactivity against the MOG34-56 peptide might be due to interaction of the two epitopes during T cell activation or by interaction of the anti-MOG24-36 Th1 cells with the anti-MOG40-48 CTL. These two possibilities were tested in three marmoset twin pairs of which one sibling was immunized with a truncated version of rhMOG that contains both epitopes in physically linkage, namely residues 20 to 50 in IFA, and the other sibling with the two epitopes physically unlinked, i.e. as a mixture of the immunogenic peptides MOG14-36 and MOG34-56 in IFA. The results show that three out of three cases immunized with the non-linked epitopes in IFA developed clinically evident EAE, while of the three cases immunized with the truncated sequence MOG20-50 in IFA, only one developed clinically evident EAE, albeit at a very late stage.

## Methods

### Animals

The marmoset monkeys selected for this study were purchased from the purpose-bred colony of the Biomedical Primate Research Centre (BPRC) in Rijswijk, The Netherlands. Individual data of the monkeys are listed in Table 1. Before inclusion in the experiment, all marmosets were subjected to a complete physical, hematological, and biochemical examination. During the study, the monkeys were under veterinary care. Monkeys in experiment were pair-housed under conventional conditions in spacious cages with appropriate enrichment and free access to food and drinking water. Padded shelter was provided on the floor. The daily diet consisted of commercial food pellets for New World monkeys (Special Diet Services, Witham, Essex, UK), supplemented with raisins, peanuts, marshmallows, biscuits and fruit.

**Table 1:** Overview of marmosets in experiment with their responses to clinical EAE and presence of CNS pathology

Exp.	Twin	Monkey	Sex	Age^a^	Sacrificed (psd)	Score 2.0	Lesions on MRI	
							WM	GM
A		M08001	F	43	114	52	+	−
		M08093	F	36	81	72	++	+
		M08119	F	33	57	54	+	−
		M09047	F	28	65	56	+	−
		M09064	F	27	57	50	+	−
		M09083	F	26	111	107	+	+
B	1	M09054	F	53	115	113	++	+
		M09055	F	53	120	120	+	−
	2	M10031	F	44	122	-	+	−
		M10032	F	44	94	91	+	−
	3	M10038	F	43	122	-	+	−
		M10039	F	43	43	40	++	+/−

*Score 2.0* ataxia or blindness, *MRI* magnetic resonance image, *WM* white matter, *GM* grey matter, *F* female, *psd* post-sensitization day

^a^Age in months at the start of the experiment

### Ethics

In agreement with the Netherlands’ law on animal experimentation, all study protocols and experimental procedures were reviewed and approved by the Institute’s Ethics Committee (BPRC) before the start of the experiment (DEC#674 and DEC#736).

### Antigens

The extracellular domain of human MOG, comprising residues 1 to 125, was expressed as an unglycosylated recombinant protein in *Escherichia coli* and purified as previously described (rhMOG) [5, 19]. Synthetic MOG peptides used for immunization (residues 20–50, 14–36 and 35–56) or for in vitro assays were purchased from Cambridge Research Biochemicals Ltd. (Cleveland, UK).

### EAE induction and clinical scoring

#### Immunization with rhMOG (experiment A)

Six unrelated marmosets were immunized with 100 μg recombinant human MOG protein (rhMOG; produced in *E. coli* at the BPRC), dissolved in 200 μl PBS and emulsified in 200 μl IFA (Difco Laboratories, Detroit, MI). Monkeys that failed to develop clinical symptoms (EAE score ≥2) within 28 days were given a booster immunization with freshly prepared rhMOG/IFA formulation.

#### Immunization with MOG peptides (experiment B)

Three marmoset twins were selected for this part of the study. One sibling of each twin was immunized twice, on days 0 and 28, with 100 μg synthetic MOG20-50 peptide, dissolved in 200 μl PBS and emulsified in 200 μl IFA. The other sibling was immunized on days 0 and 28 with a mixture of two MOG peptides 14–36 and 34–56 (50 μg of each peptide), dissolved in 200 μl PBS and emulsified in 200 μl IFA. On days 56 and 84, all siblings were immunized with 100 μg MOG34-56 peptide, dissolved in 200 μl PBS and emulsified in 200 μl IFA.

All emulsions were prepared by gently stirring at 4 °C for at least 1 h, and 400 μl was injected into the dorsal skin divided over four spots of 100 μl each, two in the axillary, and two in the inguinal region. Clinical signs of EAE were scored at a daily basis using a standard scoring system [9, 11], as 0 = no clinical signs; 0.5 = apathy, loss of appetite, altered walking pattern without ataxia; 1 = lethargy, anorexia, loss of tail tonus, tremor; 2 = ataxia, optic disease; 2.5 = monoparesis or paraperesis, sensory loss; 3 = paraplegia or hemiplegia; 4 = quadriplegia; 5 = spontaneous death due to EAE. Overt neurological symptoms were defined as score 2 or higher. At score 3, monkeys were humanely killed for ethical reasons.

### Postmortem examination

Marmoset monkeys reaching the pre-determined time of necropsy or a maximal EAE score of 2.5 were sedated by intramuscular injection of alfaxan (0.2–0.3 mg/kg) and ketamine (40–50 mg/kg) (Vétoquinol S.A., Magny-Vernois, France). After collection of the maximum volume venous blood (PBMC) into heparinized vacutainers, monkeys were euthanized by infusion of sodium pentobarbital (Euthesate®, Aphormo, Duiven, The Netherlands). At necropsy, the brain and spinal cord were removed and fixed with 4 % buffered formalin. Fixed brains were analysed with magnetic resonance imaging (MRI) as described for assessing the size and spatial distribution of lesions [20, 21]. The fixed brain and spinal cord were examined for histopathological changes [20, 21], in particular the severity of inflammation and demyelination. Secondary lymphoid organs were aseptically removed for preparation of mononuclear cells (MNC), including the spleen, axillary (ALN), inguinal (ILN), lumbar (LLN) and cervical (CLN) lymph node [21].

### MNC proliferation

MNC were isolated every 2 weeks from heparinized blood or at necropsy from the spleen, ALN, ILN, LLN and CLN, as previously described [21] and cultured with a panel of overlapping rhMOG peptides or the complete rhMOG protein (each 10 μg/ml). Proliferation was quantified by [^3^H]-thymidine incorporation and expressed as stimulation index (SI), representing the response in antigen-stimulated versus unstimulated cultures. SI values above 2 were considered positive as customary for non-human primate T-cells.

### Antibody detection

Antibody binding to rhMOG or to a panel of overlapping MOG peptides was measured in plasma samples using ELISA as previously described [21]. For detection of antibody to conformationally intact MOG expressed in myelin, marmoset CNS white matter myelin was isolated by homogenization and sucrose gradient centrifugation [22, 23]. Hereafter, 20 μl of 1 mg/ml myelin was added to pre-diluted 100 μl plasma samples in a 96-well plate, 30 min at 37 °C. After two wash steps with 180 μl 1 % BSA/PBS by centrifugation for 10 min at 4000×*g*, pellet was incubated with the secondary antibody goat-anti-monkey IgG-FITC (1:500, 100 μl/well) (Rockland, Gilbertsville, PA), 30 min at 37 °C. IgG binding to myelin particles was measured by a FACS LSRII flow cytometer using FACSDiva software (BD Biosciences). Mean fluorescence intensity (MFI) was determined of the FITC channel, and data was corrected for background staining (myelin particles incubated with the secondary antibody only). For blocking of anti-MOG IgG molecules in plasma, samples were pre-incubated for 1 h at 37 °C with a dose titration of rhMOG before it was added to the myelin particles.

### Quantitative PCR

PBMC, spleen and ALN cell suspensions were prepared for total mRNA extraction using RNeasy minikit (QIAGEN, Hilden, Germany), and subsequently, cDNA was made using RevertAid First Strand cDNA Synthesis kit (Fermentas, St. Leon-Rot, Germany) according the manufacturer’s instructions. Quantitative PCR (qPCR) was performed as described previously [21].

### Cytokines

Supernatants of PBMC, ALN and spleen cell cultures were collected after 48 h stimulation with rhMOG or a panel of overlapping MOG peptides. Supernatants were tested according to manufacturer’s instructions with commercial ELISA kits for new world monkey TNF-α, and new world monkey IFN-γ (both from U-Cytech, Utrecht, The Netherlands), and human IL-17A (eBioscience, San Diego, CA) [21].

### Statistical analysis

Data is presented as mean ± SEM. Statistical analysis was performed using the Mann–Whitney *U* test. *p* values ≤0.05 were considered significant. For the immune assays, values above the mean background ±2 SD for T-cell proliferation (stimulation index (SI) ≥2) and antibody production (fold increase ≥2 relative to pre-immune serum) were considered positive.

## Results

### Atypical EAE induced with rhMOG in IFA

**Fig. 1:**
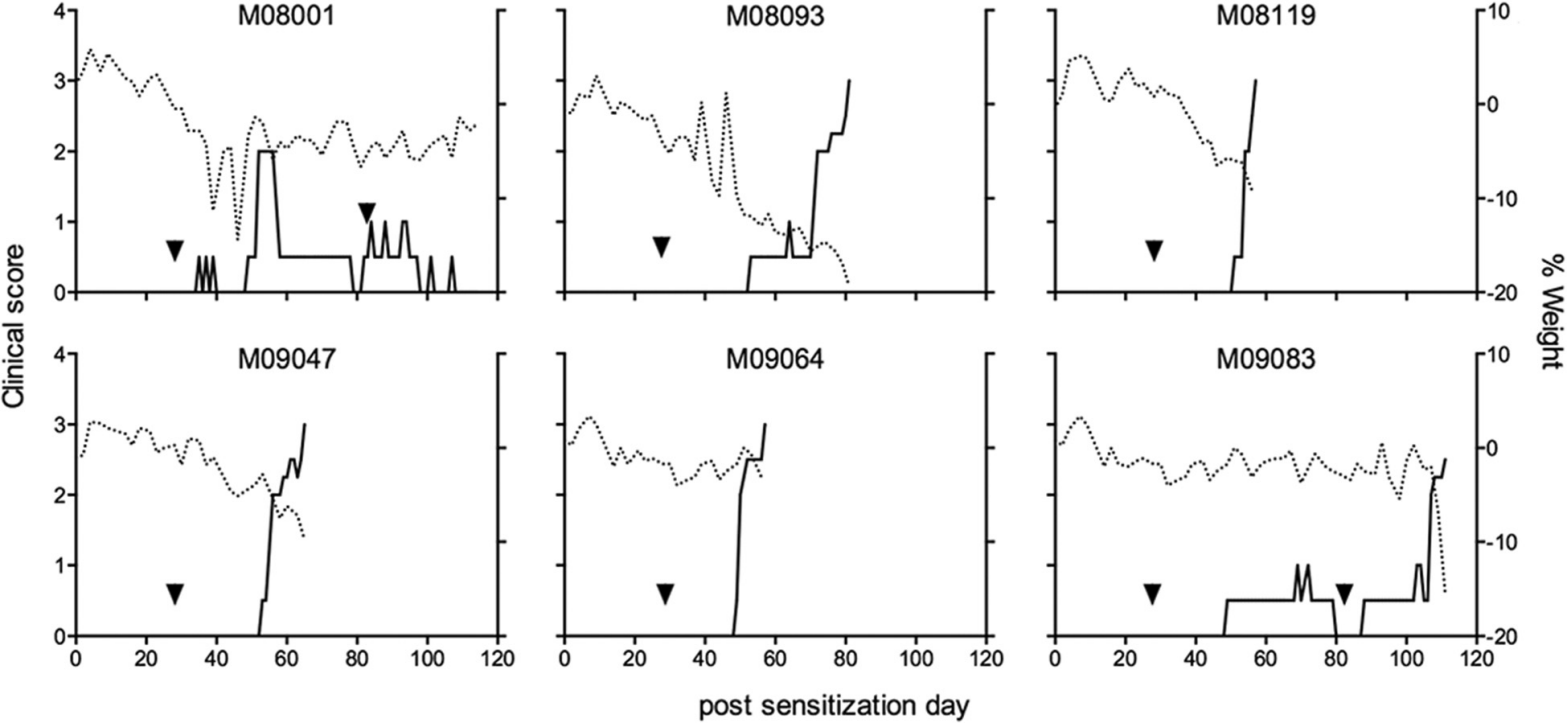
rhMOG in IFA induces clinical EAE in marmoset monkeys. Six unrelated healthy marmosets were immunized with rhMOG in IFA (post-sensitization day 0). Monkeys not developing clinically evident EAE within 28 days were given one or two booster immunizations with the same antigen-adjuvant emulsion (*arrowheads*). Clinical scores and body weights are shown for all marmosets. Clinical scores are depicted on the left *y*-axis (*solid line*) and the percentage body weight loss compared with day 0 on the right *y*-axis (*dotted line*)

Six unrelated common marmosets were immunized with rhMOG in IFA. Figure 1 shows that the clinical presentation varied between individual monkeys, likely reflecting the outbred nature of the model. Monkey M08001 displayed only a short episode of ataxia after the second immunization, but the EAE remitted thereafter. A third immunization with the same antigen preparation failed to induce a relapse; this monkey was therefore sacrificed without evident EAE symptoms. The aberrant EAE course was reflected by the body weight measurements. Four monkeys (M08093, M08119, M09047 and M09064) required only two immunizations for full development of clinically evident EAE (score 3.0; paralysis) at which stage they were sacrificed for ethical reasons. These four monkeys displayed the normal clinical EAE course, characterized by rapid accumulation of neurological deficits. Neurological symptoms typically started as ataxia (EAE score 2.0) and progressed within a few days to paresis of two hind limbs (EAE score 2.5) and paralysis of one or both hind limbs (EAE score 3.0). The sixth monkey (M09083) developed neurological signs after three immunizations and was sacrificed with EAE score 2.5 (paresis of two hind limbs). The disease presentation in case M09083 differed from the remaining monkeys as its hind limbs displayed signs of spastic paresis while the forelimbs were weakened.

### Brain lesion load assessed with MRI and histopathology assessment

Brains collected at necropsy were briefly fixed with buffered formalin and then examined with MRI for the presence of lesions in the white matter using the parameters: volume, T2 relaxation time signal (T2) and magnetization transfer ratio (MTR). White matter attenuated inversions recovery (WAIR) imaging was used for the detection of lesions in the grey matter. The 3D reconstructions of T2-weighted MR images, visualizing the spatial localization of lesions, show that the lesion load is highly variable between the six analysed monkeys. Table 2 summarizes the MRI data of the individual monkeys.

**Table 2:** Overview of post-mortem MRI parameters of each marmoset monkey of experiment A

	Animals	WM	NAWM	WM Lesion	GM	NAGM	GM Lesion
Volume (mm^3^)^a^	M08001	61.5	61.5	0.6	107.0	107.0	–
	M08093	70.3	43.6	65.1	112.8	112.7	0.6
	M08119	54.1	54.1	0.6	104.5	104.5	–
	M09047	48.1	48.1	0.3	99.9	99.9	–
	M09064	49.1	47.5	2.8	103.0	103.	–
	M09083	60.8	60.8	3.8	107.1	107.1	0.2
	Mean	57.3	52.6	12.2	105.7	105.7	0.4
	SEM	3.5	3.0	10.6	1.8	1.8	0.2
T2 (ms)	M08001	28.2	28.2	36.5	34.0	34.0	–
	M08093	35.3	33.7	35.7	39.9	39.9	37.4
	M08119	24.3	24.3	28.6	29.8	28.8	–
	M09047	29.6	29.6	36.7	36.8	36.8	–
	M09064	23.2	23.1	26.8	28.3	28.3	–
	M09083	30.9	30.9	31.2	39.1	39.1	34.9
	Mean	28.6	28.3	32.6	34.6	34.6	36.2
	SEM	1.8	1.6	1.8	2.0	2.0	1.3
MTR (%)^b^	M08001	40.6	40.6	33.2	27.6	27.6	–
	M08093	36.5	39.9	31.4	27.4	27.4	28.8
	M08119	42.0	42.0	35.5	27.8	27.8	–
	M09047	42.7	72.7	33.0	28.6	28.6	–
	M09064	44.0	44.2	37.8	28.7	28.7	–
	M09083	42.5	42.5	34.0	28.2	28.2	27.8
	Mean	41.4	42.0	34.1	28.1	28.1	28.3
	SEM	1.1	0.6	0.9	0.2	0.2	0.5

^a^Lesion volumes in GM and WM were determined in the total brain, values of WM, NAWM, GM and NAGM were only determined for a single pre-defined slice, namely the first caudal to rostral slice that showed the complete corpus callosum connection

^b^MTR values are expressed as % decrease signal intensity

**Fig. 2:**
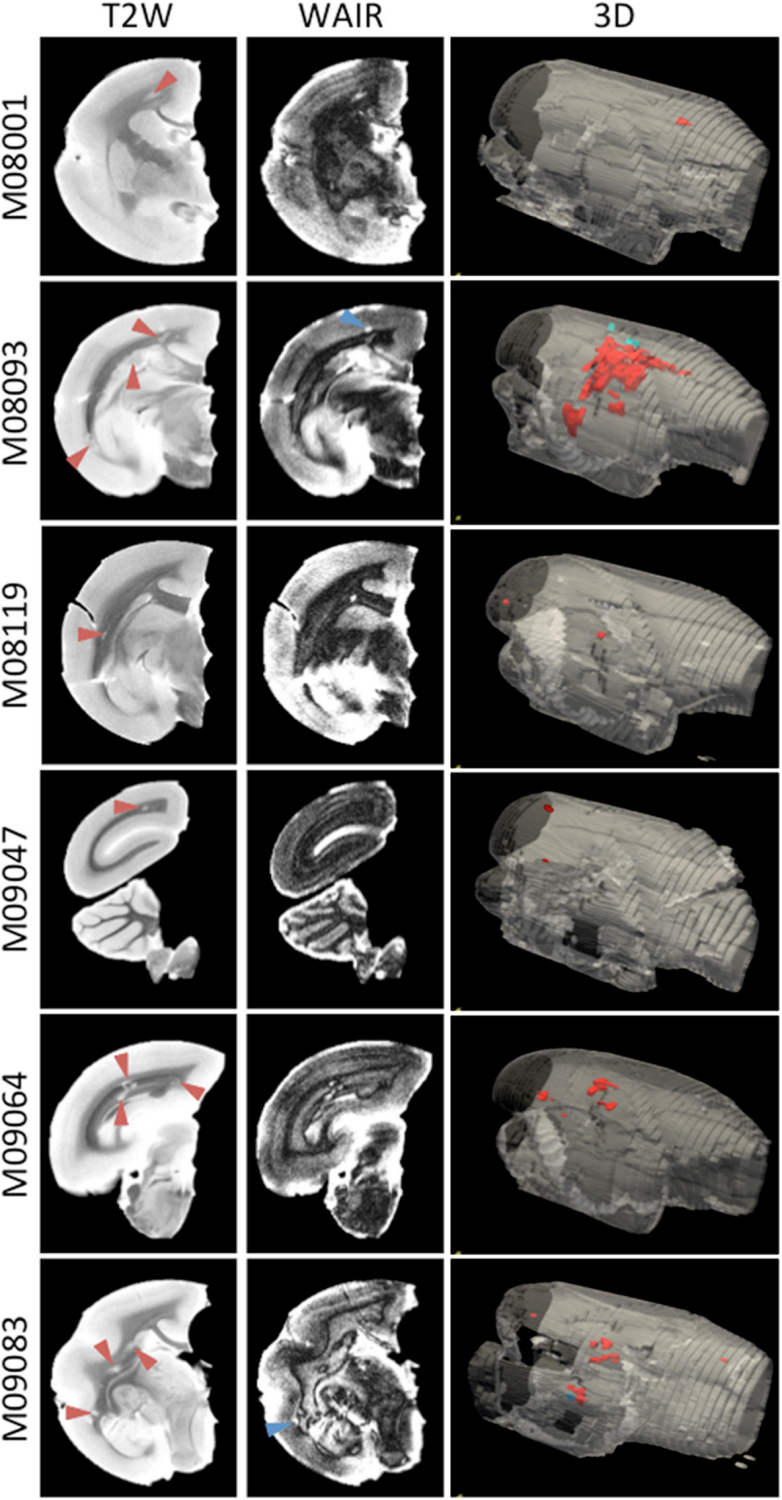
MRI images. MRI scans were made of formalin fixed brains. A representative slide of T2-weighted (T2W) and white matter attenuated inversion recovery (WAIR) images are depicted. In addition, a 3D reconstruction was made of all T2W images. The *left two columns* display typical slices through the brains on which lesions in the white (*red arrowheads*) and grey (*blue arrowheads*) matter lesions can be discerned. The 3D volume shows the total outlined volume of white (*red*) and grey matter (*blue*) lesions for each animal. Note that grey matter lesions were only observed in M08093 and M09083

The MRI scans revealed in all six cases the presence of lesions in the white matter, albeit different in size and number, while in two cases (M08093 and M09083), we found lesions also in the grey matter. In monkey M08093, the highest white matter lesion volume was detected. Figure 2 shows the presence of lesions in a selected coronal slice from each marmoset brain. The T2-weighted images (T2W) reveal lesion-like abnormalities detected in the white matter, while the corresponding WAIR images of the same slice show a grey matter lesion. Overall, the MRI-detectable brain lesion load in this new model was remarkably low compared to the model induced with rhMOG/CFA, despite the presence of similar serious neurological defects in both models.

Histopathological examination (see Table 3) confirmed the presence of moderate demyelination in the brain white matter. In all monkeys, except the case that was sacrificed without clinical EAE (M08001), a variable degree of inflammation and demyelination was detected in the spinal cord.

**Table 3:** Neuropathology quantification of the EAE model induced with rhMOG/IFA

	Spinal cord		Brain	
Monkey	Inflammation/mm^2a^	Demyelination (%)	Demyelination WM (%)	Demyelination GM^b^
M08001	0.0	0.0	0.0	−
M08093	2.9	32.0	12.0	±; Intracortical
M08119	0.3	1.0	0.2	−
M09047	0.1	5.4	6.5	±; Intracortical
M09064	0.7	7.9	1.3	−
M09083	0.4	4.2	0.1	±; Intracortical

^a^The inflammatory index of the spinal cord is defined as the average number of inflamed blood vessels/spinal cord cross sections. In total, eight slices for each animal were stained, which equals 6 cm^2^ in total

^b^Demyelination in brain GM was quantified as: + = demyelination; ± = some demyelination; − = no demyelination

### Plasma antibody levels

**Fig. 3:**
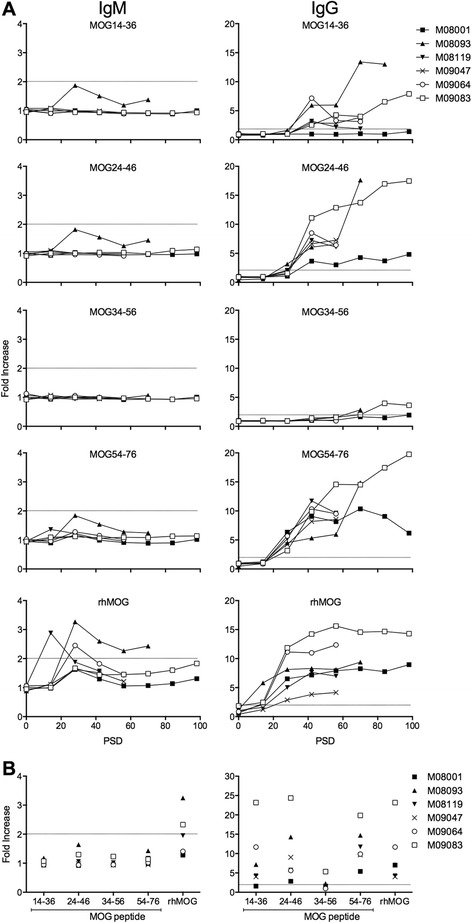
Plasma antibody levels. Plasma samples were collected longitudinally (**a**) and at necropsy (**b**) and tested for the presence of IgM (*left column*) and IgG (*right column*) antibodies binding ELISA-plate bound rhMOG or MOG peptides 14–36, 24–46, 34–56 and 54–76. Results are quantitatively expressed as fold increase relative to pre-immune plasma of the same monkeys. Fold increase ≥2.0 is considered positive. *PSD* post-sensitization day

Plasma samples were collected at 2-week interval and at necropsy. All samples were tested for the presence of IgM and IgG antibodies binding to plate-bound rhMOG or the MOG peptides 14–36, 24–46, 34–56 and 54–76 (Fig. 3). Longitudinal analysis showed that serum levels of IgM antibodies peaked between days 14 and 28 after immunization, while IgG levels increased progressively. We observed relevant binding, i.e. above the cutoff level 2, of IgM antibodies in the immune sera only to rhMOG, but to none of the MOG peptides. Elevated IgG levels were found in all animals from day 14 after immunization onward; IgG binding to rhMOG as well as to peptides 14–36, 24–46 and 54–76 was detected. The highest IgG antibody binding was measured in M09083.

Remarkably, IgG binding to the pathogenically most relevant MOG34-56 peptide could not be detected, although previous studies show that immunization with MOG peptides 34–56 in IFA elicited IgG antibodies [11].

### Antibody levels against native MOG

**Fig. 4:**
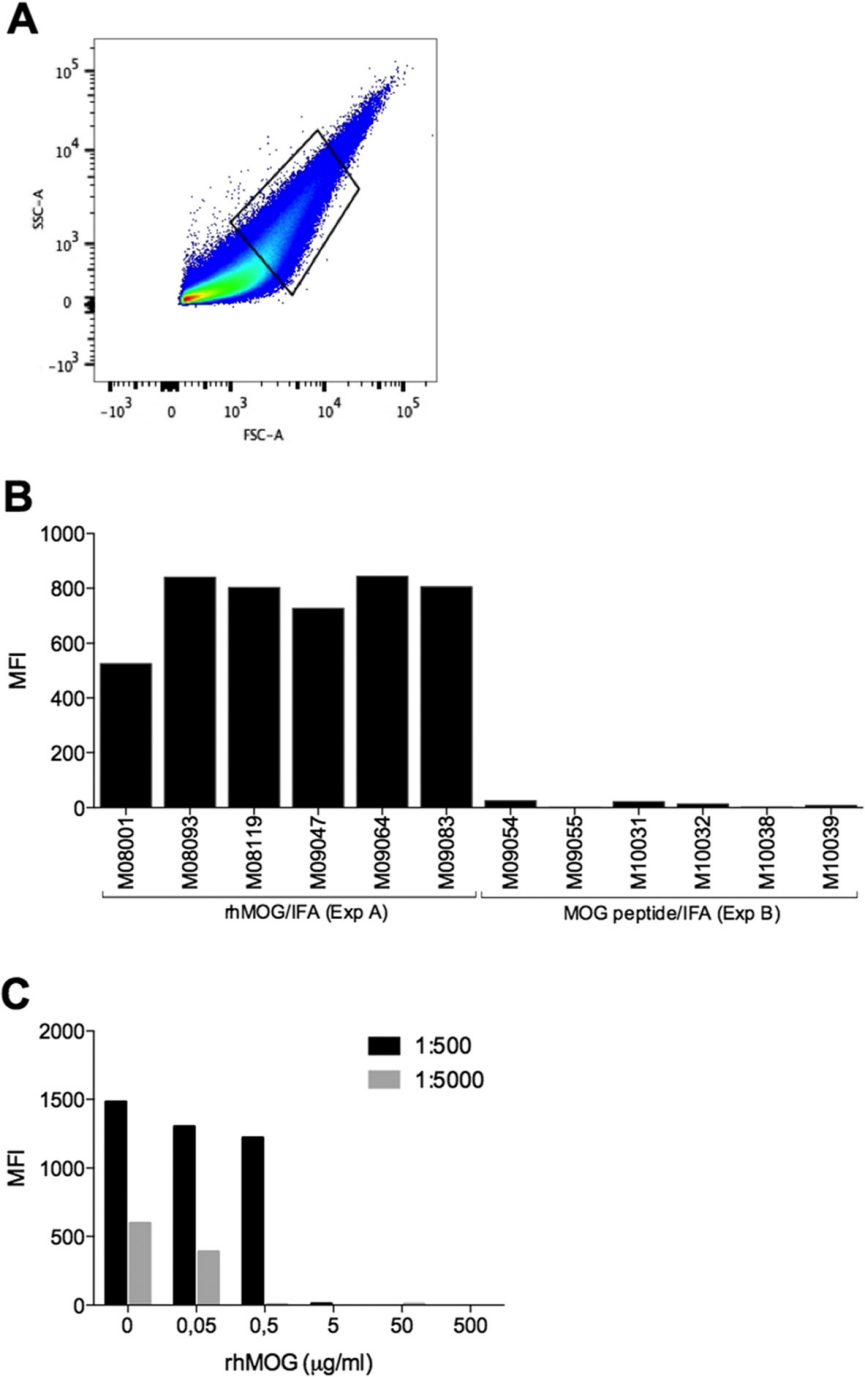
Plasma IgG binding to native MOG. Necropsy plasma samples were tested for IgG binding with healthy marmoset myelin particles. **a** Gating strategy: myelin particles were plotted at a logarithmic FSC and SCC scale. Middle-sized particles were selected to determine the mean fluorescence intensity (MFI) in the FITC channel. **b** Marmoset myelin particles were incubated with 2000× diluted necropsy plasma samples, and IgG binding was detected. IgG binding to myelin was expressed as mean fluorescence activity (MFI). **c** Plasma samples of monkey M09083 were pre-incubated in two dilutions (1:500 and 1:5000) for 1 h at 37 °C with a dose titration of rhMOG to block anti-MOG IgG molecules. The pre-incubated plasma samples were subsequently tested for residual IgG reactivity with marmoset myelin particles. All data were corrected with background staining with the secondary only

Plasma samples collected at height of the disease were tested for IgG binding to MOG in its natural configuration as it is expressed in healthy marmoset myelin. Figure 4 shows that in all animals, a relatively high plasma IgG reactivity with myelin particles was detected, even in plasma samples from monkeys M08001 and M09047, which displayed low reactivity with ELISA plate-bound rhMOG. Data in Fig. 4c depicts that plasma IgG binding to the myelin particles was blocked when plasma samples were pre-incubated with a dose titration of rhMOG. This experiment confirms that IgG binding is really directed to MOG.

### Proliferation of MNC from blood and lymphoid organs

**Fig. 5:**
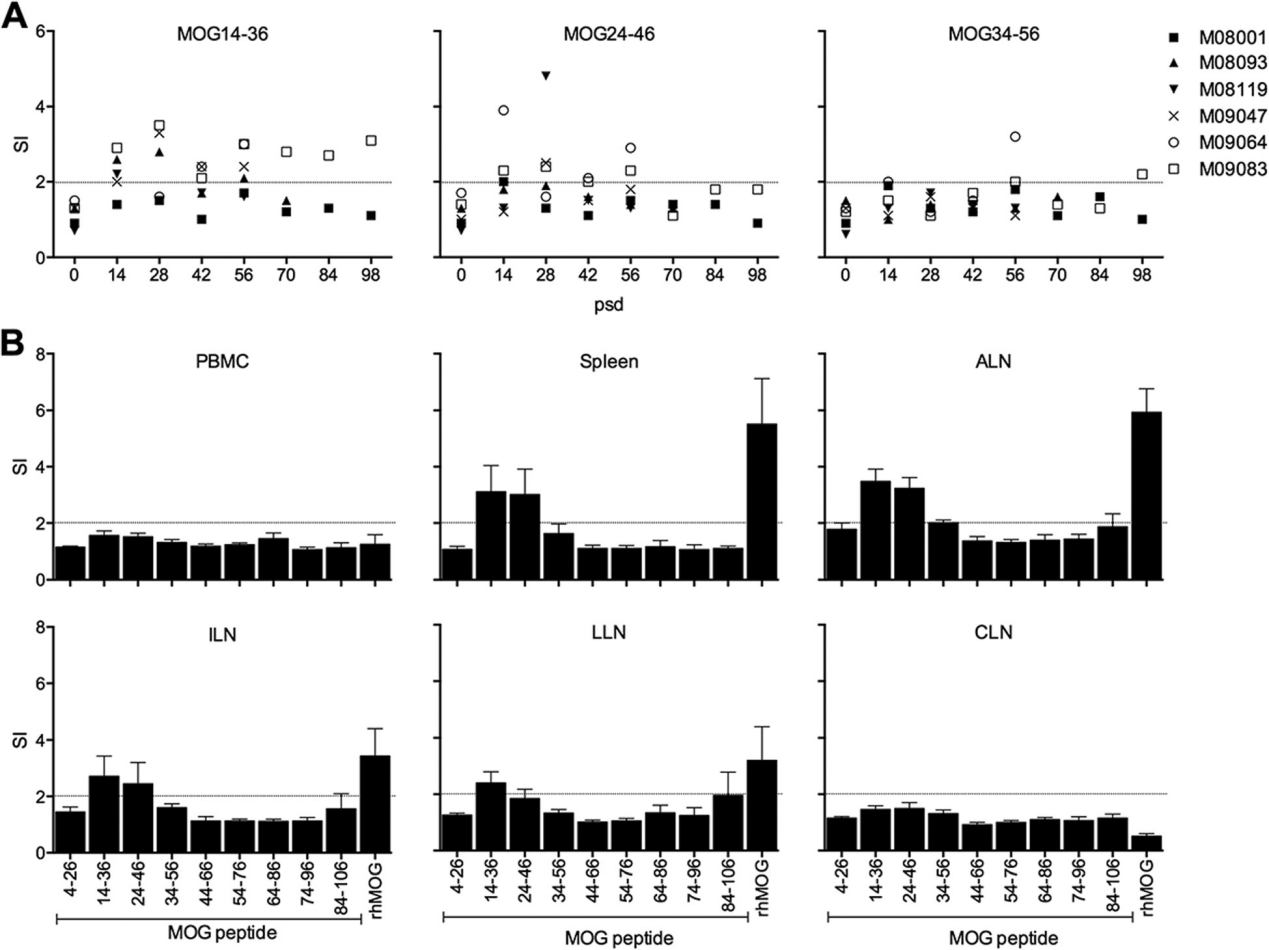
MNC proliferation against MOG antigens. **a** MNC were isolated at 2-week interval from venous blood and tested for proliferation against a panel of 23-mer overlapping MOG peptides. The positive proliferation data from individual monkeys, i.e. against MOG14-36, 24–46 and 34–56, are shown. **b** At necropsy, MNC were prepared from blood (PBMC), spleen, axillary (ALN), inguinal (ILN), lumbar (LLN) and cervical (CLN) lymph nodes and were tested for their proliferation against MOG peptides and rhMOG. Data of the six monkeys are presented as mean ± SEM. Data are expressed as stimulation index (SI) relative to unstimulated cell cultures. SI values ≥2.0 (*dotted line*) are considered positive. *PSD* post-sensitization day

PBMC were collected every 2 weeks and tested for proliferation against a set of 10-mer overlapping MOG peptides of 23 amino acids length, spanning most of the rhMOG sequence: residues 4–26, 14–36, 24–46, 34–56, 44–66, 54–76, 64–86, 74–96 and 84–106. Data are only shown for the peptides eliciting a detectable proliferative response (Fig. 5a), being peptides 14–36 and 24–46 and for 34–56. A positive, albeit low, response was detectable after post-immunization day (psd) 14 against peptides 14–36 and 24–46, which defines the previously identified CD4+ Th1 epitope MOG24-36 [7]. Just like with the serum IgG, reactivity against the peptide MOG34-56 was conspicuously absent. Figure 5b depicts the proliferation of PBMC and MNC prepared from lymphoid organs (spleen, ALN, ILN, LLN and CLN) at height of the disease (i.e. the necropsy date). The figure illustrates that at this late moment in the disease course, rhMOG reactive T cells were not detectable in the blood, but resided in the ALN and ILN that drain the immunization sites and in the spleen. Proliferation activity with a SI ≥2.0 was confined to rhMOG protein and to the overlapping peptides 14–36 and 24–46.

### Antigen-induced cytokine production

**Fig. 6:**
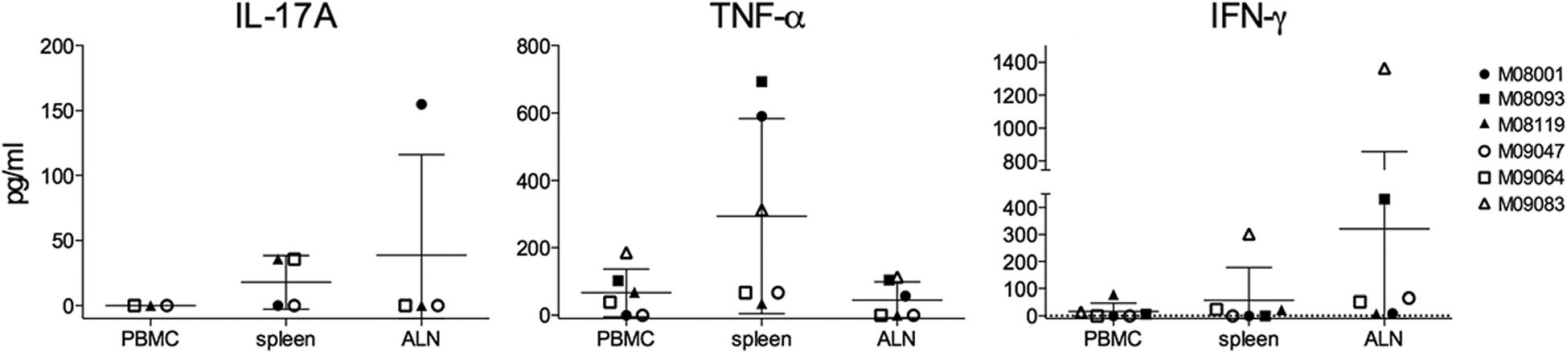
Cytokine levels in culture supernatants. MNC from the blood, spleen or ALN were cultured ex vivo with rhMOG under conditions as explained in Figure 5. After 48-h culture, supernatants were collected. Levels of IL-17A, TNF-α and IFN-γ were measured with ELISA. Data is expressed as pg/ml

For further characterization of T cell immunity in the novel EAE model, we determined the ex vivo induction of cytokines in cultures of MNC from the blood, spleen and ALN stimulated with rhMOG. The culture supernatants were assayed for the presence of IL-17A, TNF-α and IFN-γ (Fig. 6). Although IL-17A is the signature cytokine of the EAE model induced with MOG34-56 peptide in IFA [11], this cytokine was undetectable in PBMC and only at a low level in spleen and ALN. IFN-γ production was also at a low level in the PBMC and spleen, except for ALN cells of two monkeys (M08119 and M08093). In contrast to IL-17A and IFN-γ production, production of the pro-inflammatory cytokine TNF-α was found increased in all three organs, especially in the spleen. These data suggest that immunization with rhMOG in IFA may induce a Th1 prone cytokine profile, which is consistent with the dominant proliferative response of T cells against the peptides MOG14-36/MOG24-46 (Fig. 5), which define the immunodominant Th1 cell epitope MOG24-36 in the rhMOG/CFA model. In none of the cultures stimulated by the MOG peptides 14–36, 24–46, 34–56, 44–66, 54–76, 64–86, 74–96 and 84–106, we detected relevant cytokine levels (data not shown).

### mRNA transcript levels in blood and secondary lymph nodes

**Fig. 7:**
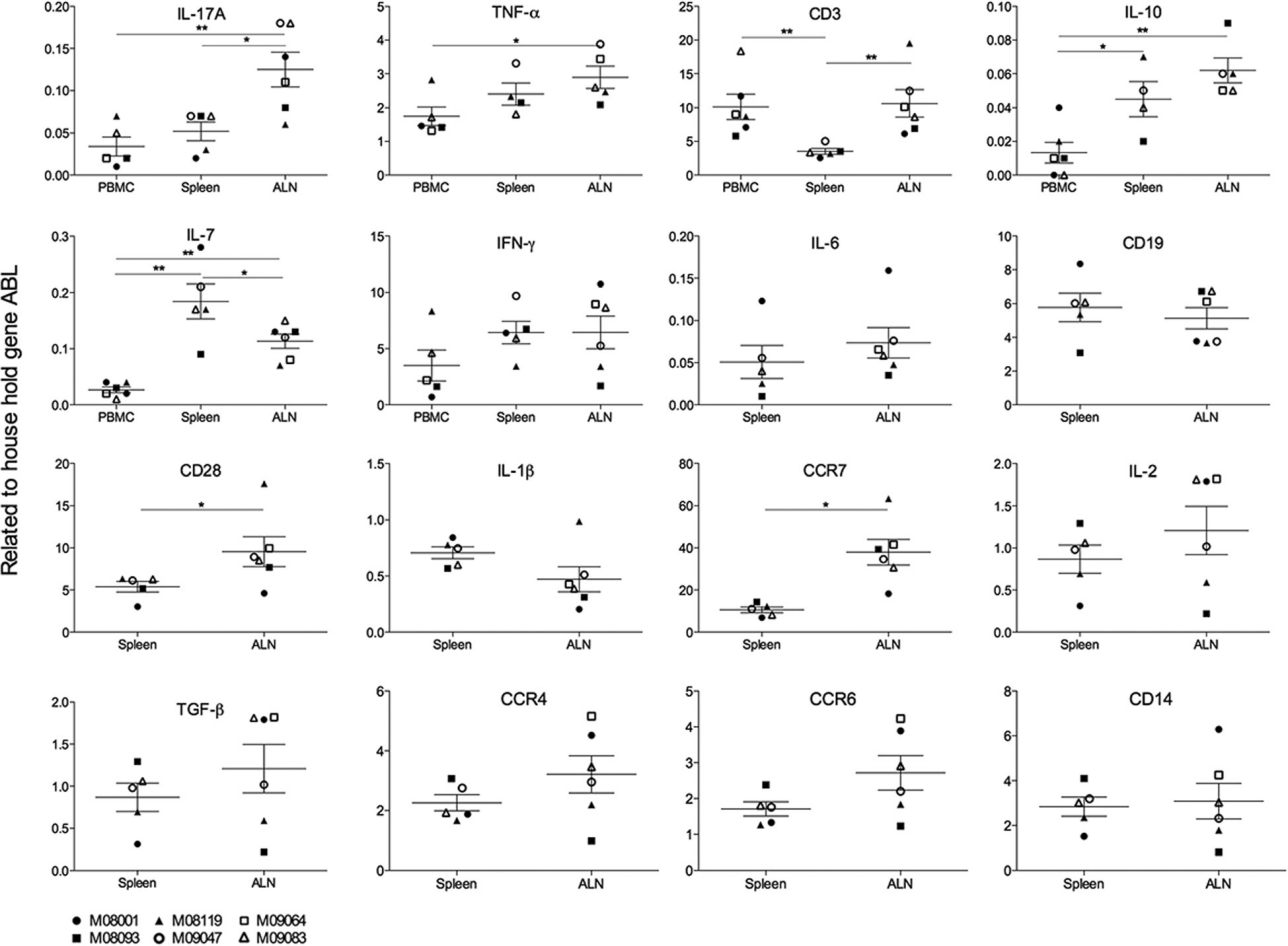
Biomarker mRNA expression levels in blood and lymphoid organs. mRNA was extracted from PBMC, spleen or ALN that were collected at necropsy. Specificity and levels of mRNA was determined with qPCR. Expression of mRNA for IL-17A, TNF-α, CD3, IL-10, IL-7 and IFN-γ was tested in all three compartments. The other markers (IL-6, CD19, CD28, IL-1β, CCR7, Il-2, TGF-β, CCR4, CCR6 and CD14) were only tested in the spleen and ALN. Data is expressed as relative to the household gene ABL

Further characterization of this new EAE model was performed by qPCR analysis of mRNA transcripts expressed in MNC from ALN and spleen. For six of the markers (IL-17A, TNF-α, CD3, IL-10, IL-7 and IFN-γ), we also analysed mRNA expression in PBMC. Figure 7 shows significant differences between compartments for the parameters IL-17A, TNF-α, IL-7, IL-10, CD3, CD28 and CCR7. For the parameters IFN-γ, TGFβ, IL-2, CCR4 and CCR6, only a trend towards different expression may be discerned. For the markers IL-6, IL-1β, CD14 and CD19, no differences were observed.

The higher expression level of CD3, CD28 and CCR7 in ALN than that in the spleen was not surprising, as the ALN drain the immunization sites. This illustrates that the immunization with rhMOG/IFA may rather induce regional immune activation than the robust systemic immune activation observed in the rhMOG/CFA model. The data also show significantly higher mRNA transcript levels for IL-17A, TNF-α, IL-10, IFN-γ and CCR7 in ALN compared to blood, although the expression of CD3 did not significantly differ. This finding, together with the CCR mRNA expression data, hints at broad T cell activation by the injection of rhMOG/IFA, including Th1 (IFNγ), Th2 (CCR4), Th17 (IL-17A, CCR6) and Tr1 (IL-10) cells. We also observed significantly higher expression of IL-7 in the spleen than in the blood and ALN.

A pathogenetically relevant feature detected in the rhMOG/CFA model was IL-7 production by B cells [24]. Figure 7 shows that CD19 mRNA levels did not differ between spleen and ALN, although production of IL-7 was highest in the spleen. This may indicate that IL-7 producing activated B cells may preferentially localize in the spleen.

### Immunization with linked and unlinked MOG epitopes in IFA

The conspicuous absence of detectable T and B cells reactivity against the pathogenetically important MOG34-56 peptide in the rhMOG/IFA model prompted us to test whether the absence of this specificity was due to interaction between the two juxtapositioned epitopes, MOG24-36 and MOG40-48. In the native MOG molecule, a N-linked glycan is attached to the MOG24-36 epitope at the Arginine 31 residue. We showed that the N-linked fucosylated glycan potentially prohibits autoimmunity against MOG by the induction of tolerogenic dendritic cells through binding to the C-type lectin receptor DC-SIGN [22], but in vivo evidence has been lacking. The observation that T cell reactivity in blood and lymphoid organs of monkeys sensitized against rhMOG/IFA was directed against the MOG24-36 epitope suggests that these T cells may have been pre-sensitized in vivo.

The juxtaposition of the two epitopes, MOG24-36 and MOG40-48 within the highly conserved 20–50 domain of the MOG molecule might enable linked suppression, as the putative Treg epitope and the Teff epitope can be simultaneously presented on the same APC. To test this possibility, we selected three marmoset twins, which due to the well-established bone marrow chimerism [25] are immunologically highly comparable. One sibling of each twin was immunized twice (days 0 and 28) with synthetic MOG20-50 peptide in IFA, in which both epitopes remained in physical linkage (=linked epitope group). The other twin siblings were immunized at the same time points with the epitopes on separate peptides, i.e. MOG 14–36 and 34–56. All immunizations were performed with IFA as adjuvant. Monkeys not developing clinical EAE were immunized twice (on days 56 and 84) with the proven encephalitogenic formulation MOG34-56 in IFA to test whether T cells capable of suppressing anti-MOG34-56 pathogenic T cells had been induced.

**Fig. 8:**
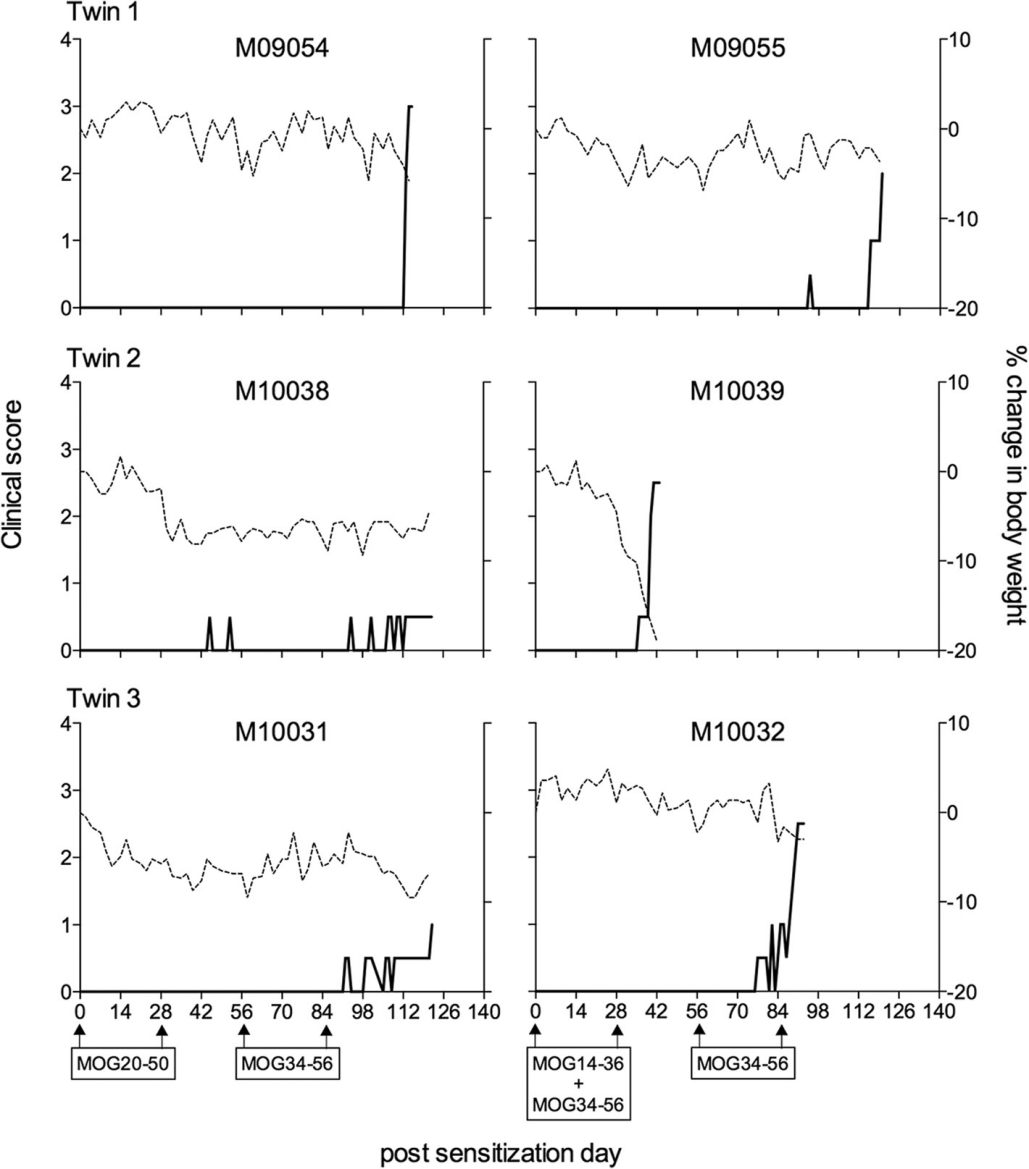
Clinical response to immunization with linked or unlinked epitopes in IFA. One sibling of each twin was immunized on days 0 and 28 with peptide MOG20-50 (*left column*); the other sibling was immunized on the same days with a mixture of MOG14-36 and MOG34-56 (*right column*). All monkeys not displaying EAE symptoms were immunized on days 56 and 84 with the encephalitogenic peptide MOG34-56. All immunizations were with peptides emulsified in IFA. Clinical scores are depicted on the left *y*-axis (*solid line*) and the percentage body weight loss compared with day 0 on the right *y*-axis (*dotted line*)

The clinical EAE scores presented in Fig. 8 show that both siblings of twin 1 developed clinical EAE at a very late stage and after the two immunizations with the encephalitogenic formulation MOG34-56/IFA. Of twin 2, the sibling immunized with the non-linked peptides (M10039) developed severe EAE at an early stage, i.e. prior to the challenge with MOG34-56/IFA, whereas the sibling immunized with the linked peptides (M10038) did not develop clinical EAE. Of twin 3, the sibling immunized with the unlinked epitopes (M10032) developed EAE shortly after the second challenge with MOG34-56/IFA, whereas the sibling immunized with the linked epitopes (M10031) did not develop clinical EAE. Interestingly, all monkeys developing EAE score of ≥2.0 showed a very fast disease progression from no symptoms or EAE score 0.5 (altered walking without ataxia) to paresis or paralysis of the hind limbs.

### Pathological parameters determined by MRI in the peptide immunized marmosets

MRI data of the three twins are summarized in Table 1, and individual data are presented in Additional file 1: Table S1. In all monkeys, white matter lesions were found, although the smallest white matter lesion volume was detected in M10031 and M10038. In twin 1, the lesion load was higher in the sibling immunized with the linked epitopes (M09054) than with the non-linked epitopes (M09055). In contrast to twin 1, we observed in twins 2 and 3 a substantially lower lesion load in the siblings immunized with the linked epitopes (M10031 and M10038) than with the non-linked epitopes (M10032 and M10039). With regard to the grey matter lesion, these were measured in two monkeys, namely in M09054 and M10039.

Taken together, the mean white matter lesion load is substantially lower in the twins immunized with the linked epitopes than those immunized with the unlinked epitopes (55.8 ± 37.1 versus 3.9 ± 2.4). Lesion characteristics, such as T2 signal intensity and MTR, did not differ between the two groups.

### Antibody responses in the linked and unlinked epitope immunized monkeys

**Fig. 9:**
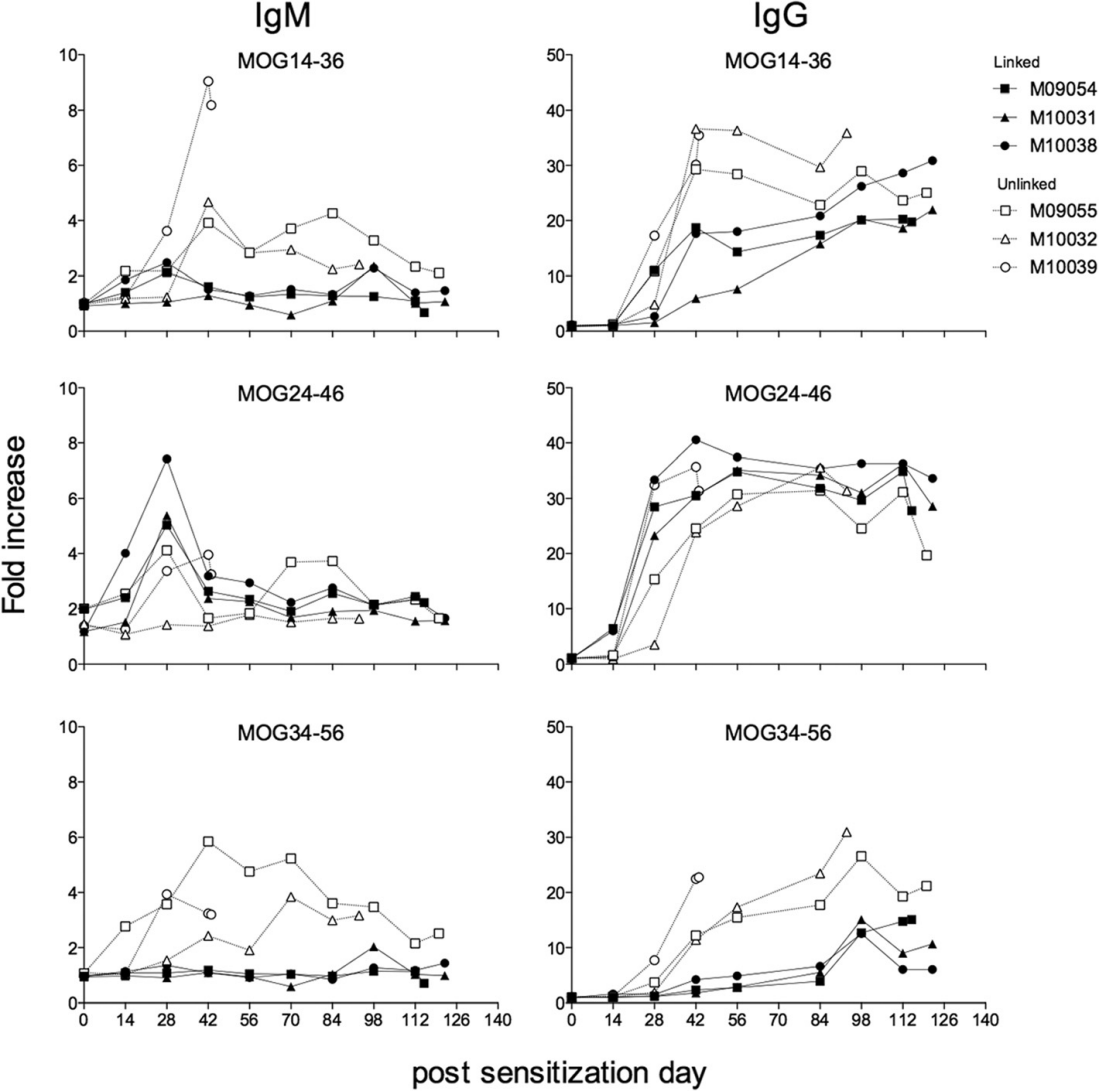
Plasma antibody levels in the linked or non-linked epitope group. Immune plasma samples were collected at day 0 and every 14 days thereafter. All plasmas were tested for the presence of IgM and IgG binding to the indicated antigens coated on ELISA plates. Results of day 70 were excluded because of technical failure. Data is expressed as fold increase relative to pre-immune plasma

Sera collected at periodic intervals were tested for binding to ELISA plates coated with rhMOG or MOG peptides. The fold increase of IgM and IgG binding relative to pre-immune sera is depicted in Fig. 9. The figure shows that in monkeys immunized with the ‘linked’ epitopes (MOG20-50), IgM levels against MOG14-36 and MOG34-56 were lower compared to the unlinked epitope group (MOG14-36 + MOG34-56). No differences of IgM binding to MOG24-56 were observed between the two groups.

IgG levels against MOG34-56 in monkeys immunized with the linked epitopes remained low for at least 80 days, while IgG levels against MOG14-36 but not against MOG24-46 were less reduced. Interestingly, IgG levels against MOG34-56 were raised after the immunization with MOG34-56/IFA. Serum levels of IgG binding MOG14-36 and MOG34-56 were clearly higher in the monkeys immunized with the unlinked epitopes.

IgM and IgG antibody levels against MOG20-50 and rhMOG showed exactly the same patterns as against MOG24-56, and no positive signal was measured against the B cell epitope MOG54-76 (data not shown). Finally, we tested plasma samples collected at height of the disease (necropsy) for the presence of IgG reactivity with marmoset myelin particles. Figure 4 shows that no such reactivity was detectable.

## Discussion

In the vast majority of EAE models, formulation of the immunizing antigen with bacterial adjuvant, such as CFA, is required for the activation of cellular and/or humoral autoimmune mechanisms. The underlying concept is that bacterial antigens relay danger signals to APC via innate receptors (TLR, NLR), which induce expression of co-stimulatory molecules that are needed for the full activation of tolerized autoreactive T cells [26]. While this ‘danger’ concept has been well validated for EAE models established in immunologically immature SPF mice [27], the question is warranted whether danger signals are (always) needed for the activation of autoreactive T and B cells present in the pathogen-educated immune system of conventionally housed primates. We are aware of only few exceptions to the rule, namely the inbred DA rat strain, in which EAE can be induced by immunization with recombinant rat MOG in IFA [28] and the outbred common marmoset in which EAE can be induced with MOG34-56 peptide in IFA [11].

The observation that CFA can be replaced with IFA in EAE induction protocols for non-human primates has important implications. First, CFA is notorious for its detrimental side effects, in particular the serious inflammatory skin reactions at the inoculation sites. Replacement of CFA for IFA thus implies a major reduction of discomfort for the animals. Second, activation signals relayed through antigen presenting cells by the mycobacteria in CFA not only awake anti-MOG T cells from their tolerized state but also cause (artificial) skewing of the T cell response towards a Th1 profile [29]. There is increasing awareness that the Th1 bias of the EAE model does not accurately reflect the complex immunopathology of MS [30] and may explain why the majority of therapies developed in the model are directed against Th1-driven inflammatory mechanisms. Many of this therapeutics were ineffective during clinical tests in relapsing-remitting MS. The anti-IL-2p40 antibody ustekinumab and the cytokine interferon-γ are illustrative examples [31, 32]. An EAE model based on IFA lacks the polarizing effect of innate immune stimulating adjuvant components and may therefore more closely reflect the natural immune response of the animals against an (injected) autoantigen.

The current study shows that six out of six marmosets immunized with rhMOG in IFA developed overt neurological signs. The data indicate that the marmoset’s immune repertoire contains pre-sensitized T and B cells, which are re-activated upon exposure to epitopes derived from rhMOG by antigen presenting cells, which have not been exposed to danger signals. Remarkably, we detected T cell reactivity with only two peptides (MOG14-36 and MOG24-46; shared epitope 24–36), while previous studies identified a second and pathogenically highly important epitope within peptide MOG34-56 (epitope 40–48) [11]. A previous study in the EAE model induced with rhMOG in CFA identified MOG24-36 as the epitope of pro-inflammatory Th1 cells, presented by the monomorphic MHC class II/Caja-DRB*W1201 allele [7]. We propose that in the rhMOG/IFA model that lacks danger signals, the autoreactive CD4+ T cells responding to MOG24-36 may differentiate into Tr1 direction.

The absence of reactivity against the MOG34-56 peptide in the current study is confusing as the 100 % EAE response to immunization with the mixture of MOG14-36 and MOG34-56 peptide in IFA (experiment B, Fig. 8) confirms the previous finding [11] that T cells capable to respond to the MOG34-56 peptide are present in the marmoset’s immune repertoire. The observation that EAE development is impaired in the fraternal siblings, which were immunized with the MOG20-50 peptide in IFA, may indicate that T cell activation against MOG34-56 is blunted when the core epitopes MOG40-48 and MOG24-36 are co-expressed in the same peptide. Note that also the IgG response against MOG34-56 was impaired in monkeys immunized with MOG20-50 in IFA (Fig. 9).

**Fig. 10:**
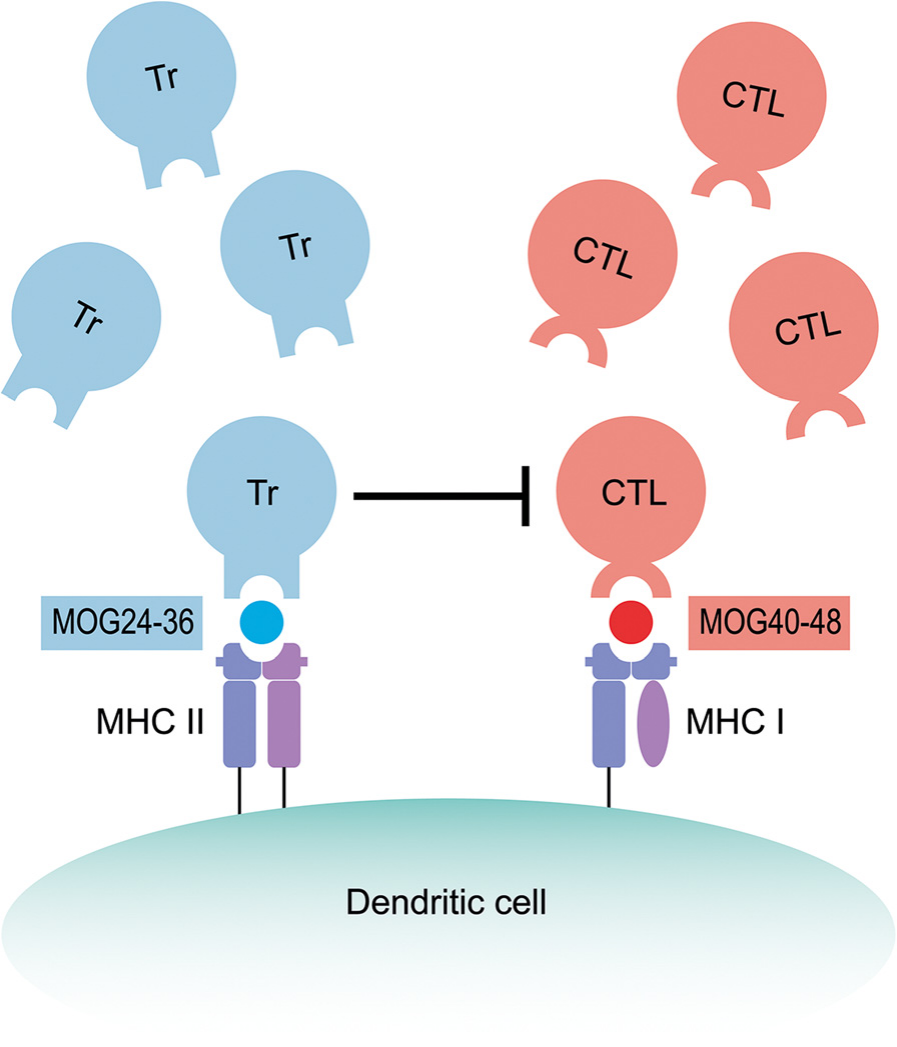
Linked suppression paradigm for the absent activation of MOG34-56-reactive T cells. We postulate that the marmoset’s immune repertoire contains pre-sensitized autoreactive T cells against two dominant MOG epitopes, i.e. the CD4 cells/Th1 epitope 24–36 (*blue triangle*) and the CTL epitope 40–48 (*red circle*), which are juxta-positioned in an evolutionary conserved region in the extracellular domain, residues 20–50. In monkeys immunized with the MOG20-50 peptide, both epitopes are processed and presented simultaneously on the APC surface. Conceptually, this implies that reactivation of CTL cells by the 40–48 epitope (*red repertoire*) may be blunted by Tr1 cells re-activated at the same APC by the MOG24-36 epitope (*blue repertoire*)

It is tempting to speculate on a possible explanation for this unexpected observation, although conclusive experimental evidence is lacking. The MOG24-36 epitope is special as the arginine residue at position 31 is the only N-glycosylation site present in MOG. We reported previously that the N-linked glycan has an important physiological function as through binding with the C-type lectin receptor DC-SIGN dendritic cells (by DC) are retained in an immature anti-inflammatory/tolerogenic state [22]. It has been shown that antigens captured by DC-SIGN are internalized and processed by the tolerogenic DC and that peptides are presented to T cells, which develop towards anti-inflammatory or regulatory functions [33]. This led us to postulate that (part of) the MOG specific T cells present in the normal repertoire may have a suppressive/regulatory function, serving the maintenance of homeostasis [34, 35]. Conceptually, it can thus be envisaged that in the monkeys immunized with the MOG20-50 peptide, the MOG24-36 and MOG40-48 epitopes are processed and presented simultaneously by the same APC (see Fig. 10). In this setting, activation of the pathogenic T cell by MOG40-48 would be blunted by the simultaneous activation of ‘putative’ regulatory cells by MOG24-36, a situation reminiscent of the linked suppression paradigm [36].

## Conclusion

In conclusion, the novel IFA-based MOG-induced EAE models in the marmoset reveal presence of pre-sensitized autoreactive T cells in the normal repertoire. The immunodominant epitopes are the same as those identified in the corresponding CFA-based models, namely residues 24–36 and 40–48. Paradoxically, we observed in the rhMOG/IFA model that activation of CD8+ CTL against MOG40-48 could not be detected. Based on previous work, we propose that (part of) the anti-MOG24-36 CD4+ T cells have regulatory/suppressive function. These may prohibit activation of highly pathogenic CTL via a mechanism reminiscent to linked suppression. The reason why the rhMOG/IFA immunized marmosets nevertheless develop EAE is unclear. The possibility that T cells against subdominant epitopes, such as 4–26 or 94–106 [7], in synergy with anti-MOG antibodies may elicit inflammatory demyelination of the CNS is subject of investigation.

## Additional file

Additional file 1: Table S1. Overview of post-mortem MRI parameters of each marmoset monkey of experiment B. (DOC 77 kb)

## Abbreviations

ALN: axillary lymph node
CFA: complete Freund’s adjuvant
CLN: cervical lymph node
CTL: cytotoxic T lymphocyte
EAE: experimental autoimmune encephalomyelitis
EBV: Epstein-Barr virus
IFA: incomplete Freund’s adjuvant
ILN: inguinal lymph node
LLN: lumbar lymph node
mAb: monoclonal antibody
MOG: myelin oligodendrocyte glycoprotein
MNC: mononuclear cells
MRI: magnetic resonance imaging
MS: multiple sclerosis
Rh: recombinant human
SI: stimulation index
Th: T helper
