# Supplementary material for: Immune profile of an atypical EAE model in marmoset monkeys immunized with recombinant human myelin oligodendrocyte glycoprotein in incomplete Freund’s adjuvant
Source: J Neuroinflammation. 2015 Sep 17;12:169. doi: 10.1186/s12974-015-0378-5 (PMC4574133; doi:10.1186/s12974-015-0378-5)
Supplement: Additional file 1: Table S1. — Overview of post-mortem MRI parameters of each marmoset monkey of experiment B. (DOC 77 kb) [file 12974_2015_378_MOESM1_ESM.doc]

Additional file Table S1. Overview of post mortem MRI parameters of each marmoset monkey of experiment B.

| **Volume (mm^3^)^a^** | **Animal** | **WM** | **NAWM** | **WM lesion** | **GM** | **NAGM** | **GM lesion** |
| --- | --- | --- | --- | --- | --- | --- | --- |
|  | **Linked** |  |  |  |  |  |  |
|  | M09054 | 41,0 | 41,0 | 10,7 | 109,6 | 109,6 | 0,24 |
|  | M10031 | 52,3 | 52,1 | 0,6 | 100,3 | 100,3 | -^c^ |
|  | M10038 | 46,5 | 46,5 | 0,4 | 95,8 | 95,8 | - |
|  | ***Mean*** | *46,6* | *46,5* | *3,9* | *101,9* | *101,9* | *-* |
|  | ***SEM*** | *2,3* | *2,3* | *2,4* | *2,9* | *2,3* | *-* |
|  | **Unlinked** |  |  |  |  |  |  |
|  | M09055 | 44,4 | 44,4 | 1,37 | 101,1 | 101,1 | - |
|  | M10032 | 46,5 | 46,5 | 5,1 | 111,6 | 111,6 | - |
|  | M10039 | 60,7 | 34,7 | 160,8 | 86,5 | 84,0 | 0,04 |
|  | ***Mean*** | *50,5* | *41,8* | *55,8* | *99,7* | *98,9* | *-* |
|  | ***SEM*** | *3,6* | *2,6* | *37,1* | *5,1* | *5,7* | *-* |

| **T2(ms)** | **Animal** | **WM** | **NAWM** | **WM lesion** | **GM** | **NAGM** | **GM lesion** |
| --- | --- | --- | --- | --- | --- | --- | --- |
|  | **Linked** |  |  |  |  |  |  |
|  | M09054 | 34,4 | 34,4 | 35,5 | 40,5 | 40,5 | 35,9 |
|  | M10031 | 30,2 | 30,1 | 35,4 | 35,6 | 35,6 | - |
|  | M10038 | 25,2 | 25,2 | 31,4 | 30,3 | 30,3 | - |
|  | ***Mean*** | *30,0* | *30,0* | *34,1* | *35,5* | *35,5* | *-* |
|  | ***SEM*** | *0,19* | *0,19* | *0,10* | *0,21* | *0,21* | *-* |
|  | **Unlinked** |  |  |  |  |  |  |
|  | M09055 | 29,6 | 29,6 | 36,3 | 32,1 | 32,1 | - |
|  | M10032 | 25,2 | 25,2 | 30,2 | 27,2 | 27,2 | - |
|  | M10039 | 28,9 | 27,6 | 33,8 | 31,4 | 31,4 | 35,4 |
|  | ***Mean*** | *27,9* | *27,5* | *33,5* | *30,2* | *30,2* | *-* |
|  | ***SEM*** | *0,10* | *0,09* | *0,12* | *0,11* | *0,11* | *-* |

| **MTR(%)^b^** | **Animal** | **WM** | **NAWM** | **WM lesion** | **GM** | **NAGM** | **GM lesion** |
| --- | --- | --- | --- | --- | --- | --- | --- |
|  | **Linked** |  |  |  |  |  |  |
|  | M09054 | 47,2 | 47,2 | 37,2 | 31,6 | 31,6 | 32,1 |
|  | M10031 | 47,4 | 47,4 | 37,8 | 33,9 | 33,9 | - |
|  | M10038 | 48,2 | 48,2 | 38,0 | 32,4 | 32,4 | - |
|  | ***Mean*** | *47,6* | *47,6* | *37,7* | *32,7* | *32,7* | *-* |
|  | ***SEM*** | *0,22* | *0,22* | *0,16* | *0,47* | *0,47* | *-* |
|  | **Unlinked** |  |  |  |  |  |  |
|  | M09055 | 47,9 | 47,9 | 38,1 | 34,3 | 34,3 | - |
|  | M10032 | 48,7 | 48,7 | 34,8 | 34,8 | 34,8 | - |
|  | M10039 | 43,7 | 45,8 | 40,0 | 32,7 | 32,7 | 32,7 |
|  | ***Mean*** | *46,8* | *47,5* | *37,6* | *33,9* | *33,9* | *-* |
|  | ***SEM*** | *1,09* | *0,61* | *1,08* | *0,45* | *0,45* | *-* |

^a^Lesion volumes in GM and WM were determined in the total brain, values of WM, NAWM, GM, and NAGM were only determined for a single pre-defined slice, namely the first caudal to rostral slice that showed the complete corpus callosum connection. ^b^MTR values are expressed as % decrease signal intensity.

^C^No GM lesions could be determined.
